# Social media use and alcohol consumption among students in Uganda: a cross sectional study

**DOI:** 10.1080/16549716.2022.2131213

**Published:** 2022-10-14

**Authors:** Edwinah Atusingwize, Maria Nilsson, Annika Egan Sjölander, John C. Ssempebwa, Nazarius Mbona Tumwesigye, David Musoke, Evelina Landstedt

**Affiliations:** a Department of Epidemiology and Global Health, Umeå University, Umeå, Sweden; b Department of Disease Control and Environmental Health, Makerere University School of Public Health, Kampala, Uganda; c Department of Culture and Media Studies, Umeå University, Umeå, Sweden; d Department of Epidemiology and Biostatistics, Makerere University School of Public Health, Kampala, Uganda; e Department of Social and Psychological Studies, Karlstad University, Karlstad, Sweden

**Keywords:** Social media, alcohol, lurking, university students, Uganda

## Abstract

**Background:**

Globally, alcohol use significantly contributes to the disease burden. Alcohol consumption in Uganda is related to several health consequences among young people, including university students. Social media is commonly used by students to share academic information and create social networks. Among young people in high-income countries, previous studies have also shown that social media use can have negative health outcomes related to alcohol use, and associated problems. To date, similar studies conducted in low- and middle-income countries are largely missing.

**Objective:**

To assess the prevalence of and associations between social media use and alcohol consumption among university students in Uganda.

**Method:**

This was a cross-sectional study among 996 undergraduate students at Makerere University. Data were collected using a questionnaire. Alcohol use in the previous 12 months was the dependent variable. The independent variable was social media use categorised as general use, alcohol-related use, and social media lurking/passive participation. Multinomial logistic regression was used to assess associations. Crude and adjusted odds ratios were reported.

**Results:**

Nearly all students (97%) used social media and 39% reported alcohol use. Regular alcohol use was significantly associated with moderate (OR = 2.22, CI: 1.35–3.66) and high level general social media use (OR = 2.45, CI: 1.43–4.20). Regular alcohol use was also associated with alcohol-related social media (OR = 6.46, CI: 4.04–10.30), and alcohol-related lurking (OR = 4.59, CI: 2.84–7.39). Similar, although weaker associations were identified for occasional alcohol use.

**Conclusions:**

Approximately four in ten students reported alcohol use in the past year, and almost all students used social media. Alcohol-related social media use was associated with occasional and regular alcohol use, with stronger associations for regular use. These findings may guide further research and present an opportunity for potential alcohol control interventions to improve health among young populations in low- and middle-income countries.

## Introduction

Alcohol consumption presents a global health concern and contributes to 5.1% of the burden of diseases such as cardiovascular diseases, cancers, hypertension and injuries [1]. Research shows a dose–response relationship – the greater the consumption, the more severe the health consequences. However, even relatively low levels of alcohol use contribute to health problems, such as HIV, TB, and some cancers [2]. Alcohol-related health consequences are particularly evident and severe in low- and middle-income countries (LMICs) [1,3,4] which could be related to different factors including inadequate alcohol regulation systems [1]. Young people is a particularly important group [1]. For instance, alcohol use among young people is associated with various health and social consequences, including violence, self-harm [5], high-risk sexual behaviours [6–8], poor school grades and death [9].

Frequent alcohol use among young people (15–24-year olds) in Eastern Africa [10], including Uganda [1], is common. Previous studies of young people in Uganda, including university students, report alcohol use prevalence of 31% to more than 50% [8,11–13]. Moreover, LMIC settings such as Uganda have a rapidly growing and poorly regulated alcohol industry and are characterised by increasing availability and affordability of alcohol for young people, as well as limited support for those experiencing alcohol-related problems [14]. Based on these observations, it is of particular importance to gain more knowledge on alcohol use and related factors in the Ugandan context. Social relationships, peer influence and peer pressure or approval have been shown to be associated with alcohol consumption among young people [15–17]. Peer influence also occurs on social media through different features, for instance the ‘likes, used as a virtual peer support for various behaviours [18].

Globally, social media is one of the growing communication channels adopted by young people. Popular social media platforms include, but are not limited to, Facebook, Twitter, YouTube, Instagram, WhatsApp, and Snapchat [19]. People use social media actively through content generation including texts, pictures, and videos. They can also react to and share content from other users. Others use social media passively or by lurking. The lurking behaviour refers to when social media users maintain their privacy while still being connected and using online communities [including social media platforms]. By following conversations or other interactions in their social media network, lurkers get access to information produced or shared by others [20,21].

Young people, including university students, use social media for various purposes such as sharing academic information and creating social networks [22,23]. However, social media can also be linked to negative health outcomes such as poor mental health [24,25], addictive behaviours and norms regarding alcohol, tobacco and other drug use [26]. Although the cause–effect relationship is unclear, alcohol-related content on social media has been found to be linked to alcohol use including hazardous drinking among youth [27], alcohol-related problems, cravings, and risk for alcoholism [28,29]. Despite considerable heterogeneity regarding measurement of alcohol-related social media and drinking behaviour in previous studies, research suggests that alcohol-related social media engagement is correlated with both greater self-reported drinking and alcohol-related problems [30]. For example, engagement with alcohol marketing content on social media have been associated with alcohol use among young people [31,32].

Social media use and its influence on behaviours, including alcohol use, can be understood through the concepts of *Connection, Comparison, Identification* and *Immerse experience* [33,34]. Connection is a central feature of social media since they provide and improve peer communication and networking. Social media also enables peer comparison through photos and peer feedback, a pivotal process of users’ online identity development. Immersive experience describes how social media provides positive, negative, immersive, and powerful experience among users [33,34]. Based on exposure to content and peer reactions or feedback on social media, in addition to a vision of self (who one is and/or wants to be), users reflect on their identity and revise it in real time. It is suggested that social media is particularly powerful in influencing young people because of features such as interactivity and portability, as well the users’ roles in creating, distributing, and consuming content of social media [35].

Existing research of alcohol and social media associations have been primarily conducted in high-income countries [19,27–40]. There is a knowledge gap concerning prevalence of social media and related alcohol consumption among young people including university students in LMICs [32]. What is generally known is that the alcohol market, alongside internet use and different social media such as WhatsApp, Snapchat, Instagram, YouTube, and Twitter are fast growing in LMICs [3]. Given the current research gap, the increasing social media use and the inadequate alcohol control interventions in LMICs, it is important to assess the relationship between social media use and alcohol consumption among young people in these settings. The present study aims to assess the prevalence of, and associations between social media use and alcohol consumption among university students in Uganda.

## Methods

### Study design and setting

This cross-sectional study was undertaken at Makerere University main campus in Kampala. Established in 1922, Makerere is the oldest university in Uganda and the East African region. Compared to other universities in the country, Makerere is the biggest and leading institution of higher education. The university consists of ten autonomous administrative units (nine constituent colleges and a School of Law), with approximately 36,000 students enrolled in undergraduate programmes (both privately and publicly funded). Located in the central division of Uganda’s capital city, the university is surrounded by various small and large commercial businesses entities such as supermarkets, retail shops, hotels, hospitals and clinics, banks, and entertainment venues including bars and restaurants. Alcohol is readily available [41] in many brands including packs of very low quantities and prices accessible and affordable to young people [41,42] such as university students.

### Participants and sampling

The study involved male and female undergraduate students. The sample size was estimated using Kish Leslie’s formula n = (Z^2^ pq)/d^2^ [43], where *n*=sample size; Z = 1.96 (standard normal deviation at 95% confidence interval); p= prevalence of alcohol use among students (using a 50% prevalence of alcohol use in a previous study) [13]); q = 1-p, q = 1–0.5 = 0.5; d=maximum error allowed (commonly 5%). Assuming a 10% non-response and a design effect of 2 to control for potential sampling errors, the minimum sample size was estimated to 844 students.

Simple random sampling technique was used to select participants from the university administrative units (9 colleges and 1 school). The target was to obtain at least 85 students per college, resulting in an estimated maximum number of 100 students per college. The college with the maximum number of listed undergraduate day programs (21) was used to calculate a sampling interval of 4.8 (i.e. 100/21 students). Every fifth student found in the vicinity/surroundings of the different colleges was approached by the research assistants, introduced to the study aims and invited to respond to the questionnaire. A total of 996 students participated in the study (1,091 students were invited, and 95 declined).

### Data collection

Data were collected between January and March 2020 by six research assistants who were recruited among recent graduates at Makerere University and had experience of data collection. Recent graduates were considered suitable because students would likely feel most comfortable responding to a peer concerning their alcohol use. Alcohol use by students can be a sensitive topic as it is generally an issue of concern due to the alcohol-related problems and their impact on the youth in the country [42]. The research assistants were trained for two days on the study objectives and how to use the data collection tool. The questionnaire was pre-tested among university students selected from three programs at Makerere (the involved programs were excluded from the final study). Following the pre-test, necessary modifications of the survey questions were made, for instance on logic sequence and wording to ensure that they were clear, simple to administer and easily understood by the respondents.

Students responded to the survey in places of their convenience, for example lecture rooms, or open spaces such as under tree shades in the compound of different colleges and halls of residences. Data were collected between 8 am and 6 pm using the KoBoCollect mobile app [44] to facilitate data collection in a population that during pre-testing showed low interest in filling in a paper-based questionnaire. The app has been successfully used in other studies [45,46]. KoBoCollect was also considered to improve data quality because of the logic of skip patterns minimising response bias. For example, students indicating no alcohol use were not presented with follow-up questions regarding alcohol use.

### Variables

#### Alcohol use/consumption

The dependent variable was alcohol use in the previous 12 months measured by an item obtained from the WHO Alcohol Use Disorders Identification Test (AUDIT) questionnaire [47]. Specifically, students were asked about how often they had a drink containing alcohol (e.g. beer, wine, vodka, gin, whiskey, brandy, rum, fermented cider, tequila, Malwa (*local brew*), or other liquor) in the previous 12 months. The question had five response options: ‘never’, ‘monthly or less’, ‘2 to 4 times a month’, ‘2 to 3 times a week’, and ‘4 or more times a week. The responses were categorised into three groups: Abstainers (‘never’ - students who had never drunk alcohol); Occasional drinking (monthly or less), and Regular drinking (2 or more times a month)—all other options combined in the previous 12 months. In the regression analysis the ‘Abstainers/never’ category was used as reference.

#### Social media use

The independent variable was students’ social media use in the previous three months. It was measured in terms of: *general social media use* (use of any media platform for any purpose); *alcohol-related social media use* (use of social media for any alcohol-related practice/activity); *general lurking behaviour*; and *alcohol-related lurking behaviour*. Lurking behaviour (general as well as alcohol-related) is more passive than general and alcohol-related social media use.

Specifically, *general lurking behaviour* was measured by asking students about how many times (or how often) they had checked their social media sites for updates or notifications (without posting, liking, commenting or sharing anything). The *alcohol-related lurking behaviour* was assessed by asking students about how many times, or how often, they had checked their social media sites for updates or notifications related to alcohol content (without posting, liking, commenting, or sharing anything). *Alcohol-related social media use* was measured by asking students about how often they had created a status update or posted on their timelines any content related to alcohol on social media sites.

G*eneral social media use* was measured by asking how often/how many times they used social media (to share updates about themselves or see updates about other users or posting any contents e.g. photos or videos) in the previous three months. Response options in all questions on social media (whether general or alcohol-related), were: ‘never’, ‘occasionally/if less than once a month’, ‘every month/monthly’, ‘every week/weekly’, ‘daily/once a day’, ‘2–9 times a day’, and ‘10 or more times a day’. Students who responded with ‘I do not know’ option (14 students altogether) were considered as ‘missing’.

General social media use was recoded into three categories: 1) High social media use (10 times or more a day); 2) Moderate social media use (2–9 times a day); and 3) Low social media use (once daily or less). The third category was a combination of different responses (daily/once a day; every week/weekly; every month/monthly; occasionally/if less than once a month; and Never). In the regressions, the ‘low social media use’ category was used as reference.

For alcohol-related social media use and lurking (general and alcohol-related lurking), the response options were categorised as ‘never’ for all those who said ‘never’ and ‘ever’ for all other response options. In the regressions, the ‘never’ category was used as reference. We categorised the alcohol-related social media use and lurking (both general and alcohol-related) into yes/no (ever/never) because these variables were heavily skewed and three categories (as in the general social media use variable) would have generated too few cases in the ‘high use’ category, thus presenting study power issues in the analysis.

*Co-variates*: Based on previous findings of associations between peer relations and alcohol use in young people [48], *alcohol use among close friends* (females and males) was included as a control variable. Similarly, *having a family member (i.e. parent/guardian or sister/brother)* who drinks was regarded as ‘indicators of home environment in terms of alcohol drinking’ which may influence alcohol behaviours of individual family members [13,49]. Demographic characteristics such as *year of study, gender, students’ main accommodation and religion*, were considered potential confounders because of their possible influence on individuals’ alcohol use [13,50]. *Parents’ occupation, and student’s involvement in work* considered as indicators of student’s financial status were also included as cofounders since it may influence affordability of alcohol [51].

### Statistical analyses

Between-group comparisons were assessed using Pearson’s chi-square tests. Variables that were significant were included in the subsequent regressions. Multinomial logistic regressions were used to assess associations between social media and alcohol use. The level of statistical significance was set at a *p*-value of 0.05. All analyses were conducted in Stata (Version 14.0) [52].

### Ethical considerations

This study was approved by the Institutional Review Board of Makerere University School of Public Health Higher Degrees Research and Ethics Committee (protocol no. HDREC 735). The study was also registered with the Uganda National Council for Science and Technology (UNCST) (research number HS849ES). Permission was also obtained from the Makerere University main administration. All participants provided informed written consent after the study objectives were explained to them. They were informed of their right to withdraw from the study at any time and that participation was voluntary. To ensure confidentiality of study participants, no personal identifiers were collected from students, and data were stored and protected by a password only accessible to the research team.

## Results

As shown in Table 1, respondents were on average 22 years old (SD = 2.43), slightly more than a half were male (54%), and mainly living off campus in rented accommodation – hostels or other rental spaces (52%). Nearly six out of ten respondents were in their first or second year of their studies. The most reported religious affiliation was Anglicans (34%) while 11% were Muslims. A majority of the respondents indicated having close male (81%) and close female (62%) friends who drank alcohol. Parental use of alcohol was reported by almost 50% of the sample (Table 1).

**Table 1.** Distribution of demographic characteristics and alcohol use.

	Total n (%)		Alcohol use n (%)		P-value
Variables	996 (100)	*Abstainer* 611 (61)	*Occasional* 228 (22.9)	*Regular* 157 (15.8)	
**Age**, Mean (SD)	22.19 (2.43)	22.08 (2.5)	22.39 (2.3)	22.36 (2.6)	**0.045**
**Gender**					**0.012**
Women	457 (45.9)	291 (63.7)	111 (24.3)	55 (12.0)	
Men	539 (54.1)	320 (59.4)	117 (21.7)	102 (18.9)	
**Year of study**					**0.119**
One	233 (23.4)	158 (67.8)	42 (18.0)	33 (14.2)	
Two	332 (33.3)	204 (61.5)	80 (24.1)	48 (14.5)	
Three and above	431 (43.3)	249 (57.8)	106 (24.6)	76 (17.6)	
**Employment Status**					**0.015**
Not Involved in work	878 (88.2)	553 (63.0)	193 (22.0)	132 (15.0)	
Involved in work	118 (11.9)	58 (49.2)	35 (29.7)	25 (21.2)	
**Parental Education**					0.245
Tertiary	624 (62.7)	370 (59.3)	144 (23.1)	110 (17.6)	
Secondary	276 (27.7)	177 (64.1)	65 (23.6)	34 (12.3)	
Primary	96 (9.6)	64 (66.7)	19 (19.8)	13 (13.5)	
**Main accommodation**					**0.103**
Campus hall	241 (24.2)	145 (60.2)	59 (24.5)	37 (15.4)	
Home	241 (24.2)	165 (68.5)	47 (19.5)	29 (12.0)	
Rented Spaces	514 (51.6)	301 (58.6)	122 (23.7)	91 (17.7)	
**Religion**					**<0.001**
Catholic	313 (31.4)	139 (44.4)	106 (33.9)	68 (21.7)	
Muslim	112 (11.2)	98 (87.5)	8 (7.1)	6 (5.4)	
Anglican	339 (34.0)	182 (53.7)	85 (25.1)	72 (21.2)	
Pentecostal and others	232 (23.3)	192 (82.8)	29 (12.5)	11 (4.7)	
**Parental occupation**					0.352
Employed/monthly salary	457 (45.9)	268 (58.6)	112 (24.5)	77 (16.9)	
Self-employed/business	426 (42.8)	272 (63.9)	95 (22.3)	59 (13.9)	
Peasant or others	113 (11.4)	71 (62.8)	21 (18.6)	21 (18.6)	
**Alcohol use among friends and family**					
Parent/guardian drinks					**<0.001**
No	536 (53.8)	423 (78.9)	71 (13.3)	42 (7.8)	
Yes	460 (46.2)	188 (40.9)	157 (34.1)	115 (25.0)	
Sister/brother drinks					**<0.001**
No	616 (61.9)	464 (75.3)	108 (17.5)	44 (7.1)	
Yes	380 (38.2)	147 (38.7)	120 (31.6)	113 (29.7)	
Close female drinks					**<0.001**
No	380 (38.2)	318 (83.7)	43 (11.3)	19 (5.0)	
Yes	616 (61.9)	293 (47.6)	185 (30.0)	138 (22.4)	
Close male drinks					**<0.001**
No	189 (19.0)	172 (91.0)	15 (7.9)	2 (1.1)	
Yes	807 (81.0)	439 (54.4)	213 (26.4)	155 (19.2)	

Significant associations (*p* value, <0.05) in bold.

Alcohol use in the previous 12 months was reported at 39% (n = 385) indicating both occasional (22.9%) and regular (15.8%) use (Tables 1 and 2). Nearly all students used social media (n = 969; 97%). Among students who used social media, nearly six out of ten used it more than once per day (24% used it 10 or more times per day, 33% reported 2–9 times per day). Nine out of ten students reported general social media lurking behaviours, while alcohol-related lurking behaviour and alcohol-related social media use were reported by approximately 23% and 26% respectively. Based on comparisons presented in Table 1, the following variables were subsequently included in the multinomial regression analyses as control variables: age, student involvement in work/employment status, religion, gender, parental (guardian) drinking, sister or brother drinking, and close male and female friend drinking.

**Table 2.** Distribution of social media and alcohol use.

Social Media Use	Total		Alcohol use n (%)		P-value
	996 (100)	*Abstainer* 611 (61)	*Occasional* 228 (22.9)	*Regular* 157(15.8)	
**General social media use**					**0.010**
Low Social Media use (Once daily or less)	415 (43.0)	275 (66.3)	93 (22.4)	47 (11.3)	
Moderate Social Media use (2–9 times/day)	321 (33.2)	186 (57.9)	76 (23.7)	59 (18.4)	
High Social Media use (10 or more times/day)	230 (23.8)	128 (55.7)	55 (23.9)	47 (20.4)	
**Alcohol related social media use**					**<0.001**
Never	714 (74.1)	506 (70.9)	156 (21.9)	52 (7.3)	
Ever	250 (25.9)	80 (32.0)	70 (28.0)	100 (40.0)	
**Lurking behaviour-general**					**0.182**
Never	52 (5.4)	38 (73.1)	9 (17.3)	5 (9.6)	
Ever	912 (94.6)	551 (60.4)	215 (23.6)	146 (16.0)	
**Lurking behaviour-alcohol related**					**<0.001**
Never	745 (77.0)	511 (68.6)	157 (21.1)	77 (10.3)	
Ever	223 (23.0)	79 (35.4)	69 (30.9)	75 (33.6)	

Significant associations (*p* value, <0.05) in bold.

Generally, alcohol use was significantly associated with general social media use, alcohol-related media use, and alcohol-related lurking behaviour (Table 3). In the crude model for occasional drinking versus abstainers, general social media use and general lurking behaviour were not significant. On the other hand, alcohol-related social media use, and alcohol-related lurking behaviour were significant. The significant association was retained in the adjusted model, although weaker; (OR = 1.72, CI: 1.13–2.61) for alcohol-related social media use, and OR = 2.47, CI:1.60–3.81 for alcohol-related lurking behaviour.

**Table 3.** Crude and adjusted associations between social media and alcohol use. Multinomial logistic regressions using Abstainer as reference category. Odds ratios with 95% confidence intervals. Significant associations in bold.

	Crude		Adjusted*	
Social Media Use	*Occasional*	*Regular*	*Occasional*	*Regular*
	OR (CI)	OR (CI)	OR (CI)	OR (CI)
**General social media use**				
Low Social Media use (Once daily or less)	1.00	1.00	1.00	1.00
Moderate Social Media use (2–9 times/day)	1.21 (0.85–1.72)	**1.86** (1.21–2.84)	1.32 (0.88–1.99)	**2.22** (1.35–3.66)
High Social Media use (10 or more times/day)	1.27 (0.86–1.88)	**2.15** (1.36–3.39)	1.26 (0.80–1.98)	**2.45** (1.43–4.20)
**Alcohol related social media use**				
Never	1.00	1.00	1.00	1.00
Ever	**2.84** (1.97–4.10)	**12.16** (8.07–18.32)	**1.72** (1.13–2.61)	**6.46** (4.04–10.30)
**Lurking behaviour-general**				
Never	1.00	1.00	1.00	1.00
Ever	1.65 (0.78–3.47)	2.01 (0.78–5.21)	1.22 (0.54–2.77)	1.14 (0.39–3.36)
**Lurking behaviour-alcohol related**				
Never	1.00	1.00	1.00	1.00
Ever	**2.84** (1.97–4.11)	**6.30** (4.24–9.37)	2.47(1.60–3.81)	**4.59** (2.84–7.39)

*Adjusted for parental drinking, sister or brother drinking, close female friend drinking, close male friend drinking, gender, age, student involvement in work, and religion.

Regarding regular drinking, all social media variables except general lurking behaviour were significant in both crude and adjusted models. Higher frequencies of general social media use had a stronger association with regular drinking, (i.e. OR = 2.45, CI:1.43–4.20 for high social media use, compared to OR = 2.22, CI: 1.35–3.66 for exposure to moderate social media in the adjusted model). Concerning students who reported alcohol-related social media use, the odds of being regular drinker versus abstainer was 6.46 times higher compared to those who never reported alcohol-related social media use (OR = 6.46, CI: 4.04–10.30). For those who reported alcohol-related lurking behaviour, the odds of being regular drinker versus abstainer was 4.59 times higher compared to those who never reported alcohol-related lurking behaviour (OR = 4.59, CI: 2.84–7.39).

## Discussion

The current study focused on possible links between social media use and alcohol consumption among students at Makerere University. The findings contribute to a preliminary understanding of the relationship between social media use and alcohol consumption among young people in a LMIC setting with limited alcohol industry regulation, and where alcohol is easily accessible and affordable by the youth [42,53]. Alcohol use during the past 12 months was reported by 39% of the students and nearly all students used social media, although alcohol-related social media use was less common. Overall, social media use was associated with alcohol use with stronger associations for regular alcohol use and alcohol-related social media use.

Similar to studies from high-income countries [19,28,29,36], we found that social media use, and especially alcohol-related social media practices, were associated with alcohol use, particularly regular alcohol consumption among students. The identified dose–response relationship confirms previous Canadian research – the greater daily social media use, the higher the odds of regular alcohol use [29]. The results also indicate that social media use may have no major effect on occasional alcohol consumption except when purposely used to access or share alcohol-related content. In line with theory on the behavioural impact of social media use [33], it is possible that students who drink alcohol might search for alcohol-related information on social media or use social media to identify and connect [33] with friends who also consume alcohol.

These results can also be understood in relation to previous research indicating that the content and messages students encounter on social media may influence them to continue consuming alcohol [38] and that high alcohol consumption and high exposure to alcohol-related social media use may reinforce each other [39,54]. This further highlights the relevance of recognising the plausible behavioural impact of comparison [33]—social media users are presented with the opportunity to compare own drinking behaviours with those of peers as commonly depicted on social media. For instance, according to Norwegian students, exposures to alcohol positive social media posts were more commonly reported than exposure to content with a negative valence of alcohol [40]. In another study, pro-alcohol-related content (positive sentiment towards alcohol use) on Twitter outnumbered anti-alcohol tweets by more than ten times [54]. Alcohol-related posts of youth often depict alcohol in a positive social context including photos of happy people socialising and holding bottles which receive more likes and comments than non-social posts (such as posts without ‘people’) [55]. Exposure to such alcohol content may result in alcohol use or at least reinforce existing alcohol use among users that identify as such [33]. For instance, posting or sharing positive aspects of alcohol may reinforce the sharers’ alcohol use through encouraging maintenance of their alcohol use status or increasing it [39], based on comparison and identity development concept [33].

This study also explored the association between lurking behaviour on social media and alcohol use which has not been explicitly explored in previous research. The findings showed that students frequently reported general lurking behaviour on social media and that alcohol-related lurking was associated with alcohol use (both occasional and regular drinking). This association could be understood in relation to the ways alcohol companies market their products on social media with persuasive targeted messages [31,56]. Alongside this development, an increasing amount of time is used by people on social media to search for information about products and services, as well as communicating their experiences and engagement with companies [57].

Importantly, the prevalent social media use and lurking practices also represent potential opportunities for social media-based interventions to prevent alcohol consumption among university students in order to promote and protect health. Such interventions could reach both alcohol users and abstainers, regardless of whether they are involved in production of some content such as posts, comments, or other visible reactions on social media.

### Strengths, limitations and suggestions for future research

One of the study strengths is that the research assistants were familiar with the Makerere setting because they were recent graduates at the university. This was beneficial in enhancing a peer-to-peer level of interaction during data collection, thereby reducing the risk of response bias. Additionally, by controlling for potential confounders of alcohol use including drinking behaviours among parents, siblings, and close friends, the identified associations between social media use and alcohol use are robust.

A limitation of the study is that the reported alcohol use could have been under-estimated if students’ responses were affected by social-desirability bias [58]. Relatedly, compared to their own ratings, the students in this study indicated that alcohol consumption was more common among their friends (both male and female). The results also show that having friends who drink alcohol was significantly associated with own alcohol use. As previously noted, peer influence is associated with alcohol consumption among young people [13,48]. It is likely that such peer effects were at play also among students in this study, which possibly suggests a higher level of alcohol use among participants than what was reported. It is also important to note that alcohol use was higher in this study compared to some previous research in the region [11,12,51], whereas lower than reported in studies from high-income countries [30,38]. However, some studies conducted in Uganda identified higher levels of alcohol use [8,13,59] than indicated in our results. There is, therefore, a need for further research to get a clearer understanding of alcohol consumption patterns among young people in Uganda.

This study is based on a sample of students from only one university, which influences the representativeness of our findings. Another limitation is related to the measures we used to assess social media use; due to the absence of more standardised social media use measures, the comparability with other studies is somewhat limited. However, we based our measures on previous studies published within the field [30]. Nevertheless, valid and reliable measures of social media use in relation to alcohol use needs to be developed in future research [36].

Similar to previous findings on increasing social media use [57], almost all students used social media, including lurking behaviour. By including lurking behaviour, we explicitly show two different practices of social media use – the passive (lurking) and the active social media use. This distinction is important and clearly shows the different exposures young people can have on social media as opposed to considering social media use as a lumpsum factor. An understanding of who practices lurking, versus active social media use is essential in different aspects including the design, implementation and evaluation of alcohol control programmes, or further exploration of the impact of different social media markets on young people. The reasons why alcohol-related lurking, and not general social media lurking, was associated with alcohol use among students also need to be explored further. The four behavioural concepts of connection, comparison, identification and immerse experience developed to capture how social media like Facebook becomes influential) [33–35], could be explored further in order to unpack in more detail the role of active versus passive/lurking behaviour on social media.

Being a cross-sectional study, no causal relationship between social media and alcohol use could be established. In other words, the relationship between alcohol-related social media use and alcohol consumption is likely to be reciprocal, and further research is needed to explore in-depth the direction, processes and related theoretical underpinnings of how social media and alcohol use are linked. Obviously, this would call for other methodological designs and approaches such as in-depth interviews with students. Future research questions could explore; how alcohol-related decisions of young people are influenced by social media use considering its fast-changing landscape; how and what role does alcohol adverts on social media play? Future studies could also comprehensively explore how alcohol consumption has changed over the past decade in light of the changing trends in social media use. Importantly, our results could inform future research to understand in-depth, the nature of the observed alcohol and social media relationship, and how social media and related concepts of connection, comparison, identification and immersive experience [33,34] may contribute to shaping alcohol-related behaviours and potential interventions in the LMIC context.

## Conclusion

The prevalence of alcohol use was relatively high among university students. Almost all of them used social media and lurking was a common practice. More than one-fourth of students reported alcohol-related social media use. Moderate and high general social media use, alcohol-related social media use, including alcohol-related lurking, were associated with alcohol use, especially regular drinking. The association with alcohol use was stronger for alcohol-related social media. Our study provides valuable information that could guide further research about social media use and alcohol consumption. The results are also useful when exploring potential interventions to reduce alcohol consumption among university students in Uganda and LMIC settings.

## Paper context

This study provides evidence on association of social media and alcohol use by university students in Uganda. Our findings indicate that students’ regular alcohol use was significantly associated with their moderate and high general social media use. Additionally, students’ alcohol use (occasional, and regular) was associated with alcohol-related social media, with stronger associations for regular users. These findings may be similar in other universities in Uganda and other low- and middle-income countries with similar contexts.
